# PIYAS-Proceeding to Intelligent Service Oriented Memory Allocation for Flash Based Data Centric Sensor Devices in Wireless Sensor Networks

**DOI:** 10.3390/s100100292

**Published:** 2009-12-30

**Authors:** Sanam Shahla Rizvi, Tae-Sun Chung

**Affiliations:** Information and Computer Engineering, Ajou University, San 5 Woncheon-dong Yeongtong-gu, Suwon 443-749, Korea; E-Mail: tschung@ajou.ac.kr

**Keywords:** wireless sensor networks, intelligent techniques, data organization, query processing, memory management

## Abstract

Flash memory has become a more widespread storage medium for modern wireless devices because of its effective characteristics like non-volatility, small size, light weight, fast access speed, shock resistance, high reliability and low power consumption. Sensor nodes are highly resource constrained in terms of limited processing speed, runtime memory, persistent storage, communication bandwidth and finite energy. Therefore, for wireless sensor networks supporting sense, store, merge and send schemes, an efficient and reliable file system is highly required with consideration of sensor node constraints. In this paper, we propose a novel log structured external NAND flash memory based file system, called *Proceeding to Intelligent service oriented memorY Allocation for flash based data centric Sensor devices in wireless sensor networks* (*PIYAS*). This is the extended version of our previously proposed *PIYA* [1]. The main goals of the *PIYAS* scheme are to achieve instant mounting and reduced SRAM space by keeping memory mapping information to a very low size of and to provide high query response throughput by allocation of memory to the sensor data by network business rules. The scheme intelligently samples and stores the raw data and provides high in-network data availability by keeping the aggregate data for a longer period of time than any other scheme has done before. We propose effective garbage collection and wear-leveling schemes as well. The experimental results show that *PIYAS* is an optimized memory management scheme allowing high performance for wireless sensor networks.

## 1. Introduction

The continuous improvement in hardware design and advances in wireless communication have enabled the deployment of various wireless applications. Wireless sensor network (WSN) applications become essential tools for monitoring the activity and evolution of our surrounding environment. The examples of WSN applications include environmental and habitat monitoring, seismic and structural monitoring, surveillance, target tracking, ecological observation, and a large number of other applications.

In WSNs, monitoring can be deployed by the following three techniques: first, each sensor node transmits its generated data to a sink node immediately [2]. This approach is referred as *Sense and Send*. Second, every sensor node aggregates its own generated data and data coming from its children nodes and then sends to its parent node [3]. This scheme is called *Sense, Merge and Send*. Third, each sensor node stores its own generated data in its local memory. The data aggregate and are sent to the sink node when it is queried [4]. This approach is called *Sense, Store, Merge and Send*.

Currently the advanced applications follow the third approach mentioned above. They store the sensor data in local on-chip and/or off-chip flash memory and perform in-network computation when required [1,5,6]. Such in-network storage approach significantly diminishes the energy and communication costs, and prolongs the lifetime of sensor networks. As a result, many techniques in the areas of data centric storage, in-network aggregation and query processing in WSNs have been proposed.

We compare our current research *PIYAS* with previous schemes as shown in Table 1. Matchbox [7], the first file system for sensor nodes, provides only the append operation and does not allow the random access of data for modification. It does not offer built-in features for device life efficiency in terms of wear-leveling. It has small size code and occupies reduced footprints that rely on the number of open files. ELF [8] claims to outperform the Matchbox by higher read throughput and random access of data by timestamps. Like Matchbox and ELF, Capsule [9] is also a limited internal memory technique. It claims to outperform ELF in terms of energy efficiency. MicroHash [10] is an external large memory-centric approach. It appends the data in time series and uses the hash index structure for answering queries. It suffers from the need for extra I/O operations to maintain the huge metadata. However, neither of the four previously discussed approaches consider the data life efficiency in terms of in-network data persistence as they simply erase the data to provide space for new data when the is memory exhausted. Storage efficiency in terms of optimal memory bandwidth utilization is also not guaranteed in the previous schemes as a small amount of data consumes a complete memory page where remaining bytes remain un-used. Therefore, our proposed *PIYA* and *PIYAS* schemes provide long-term in-network data availability by retaining data in form of raw and aggregate data, and provide optimal utilization of memory space by gathering data in main memory buffers. The data flush in the flash memory when the data of one complete page becomes available, except in exceptional cases where the sensor stops sensing and switches to its sleep mode. Plus, we offer high throughput with various natures of queries. Furthermore, our current research *PIYAS* prolongs device life in terms of wear-leveling, plus offers higher energy efficiency.

Even though a recent study [11] shows that flash storage is two orders of magnitude cheaper than communication and comparable in cost to computation plus the fact that flash memory offers many other advantages in terms of large size and reliable storage, its special hardware read, write and erase characteristics impose design challenges on storage systems [12] (discussed in detail in Section 2.2). To overcome the problems of flash memory, the storage management techniques developed for disks may not be appropriate for flash. Therefore, to make flash media useful for sensor environments and to efficiently satisfy the network business goals and requirements relevant to sensor data storage, an efficient and reliable data management scheme is highly required.

In this paper, we propose the log-structured external large NAND flash memory based file system called *Proceeding to Intelligent service-oriented memorY Allocation for flash based data centric Sensor devices in wireless sensor networks (PIYAS)*. This is the enhanced version of *PIYA* [1]. We highlight three main problems and we aim to achieve their solutions accordingly.

- First, when flash space becomes exhausted and there is no space remaining for further data storage, the system selects the victim block for garbage collection and the data in the victim block is simply erased and then the future queries cannot access this data as in [6–10]. This generates a data failure for user applications. Therefore, to address this issue, an effective data organization policy is required to provide long term in-network data availability.
- Second, the system initialization time and size of mapping structure increases with the size of stored data and flash media as in [7,10], but size of SRAM does not follow the trend of increasing size of flash memory. Thus, for instant mounting, a reliable and small size mapping structure is necessary with consideration of limited SRAM constraints of sensor nodes.
- Third, to entertain the read intensive conditions, reading of the entire media at the time of query responses becomes a big overhead with the increasing size of data and flash capacity as in [10]. Hence, an efficient query processing framework is desired to effectively satisfy the business needs.

In this paper, beside above given goals, we aim to achieve the following additional objectives to optimize our idea:

- Minimize reads, writes and erases to secure energy,
- Effective garbage collection, and
- Reliable wear-leveling scheme.

The remainder of this paper is organized as follows. We review the background of system architecture of sensor node plus flash memory characteristics are explained in Section 2. Section 3 describes the proposed *PIYAS* scheme. Experimental results are discussed in Section 4. Finally, Section 5 presents the conclusions.

## 2. Background

### 2.1. System Architecture of Sensor Node

**Figure 1.**
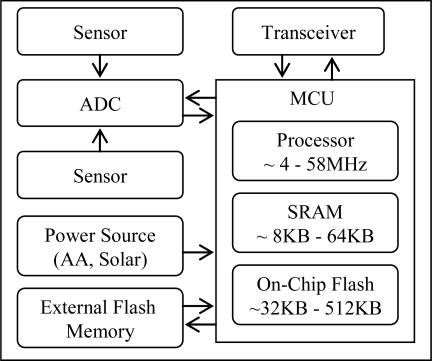
Sensor node architecture.

The architecture of the wireless sensor node consists of a microcontroller unit (MCU) that interconnects a data transceiver, sensors, along with analog-to-digital converters (ADCs), an energy source, and an external flash memory, see Figure 1. The MCU includes a processor, a static RAM (SRAM), and on-chip flash memory. The processor increases efficiency by reducing power consumption. It runs at low frequency (∼4–58 MHz) and further saves energy while the node is in standby or sleep mode. The low power microcontrollers have limited storage, typically less than 10 KB of SRAM, mainly for code execution. However in latest generation of sensors [5], it also uses it for in-memory buffering. The limited amount of on-chip flash memory provides a small non-volatile storage area (∼32–512 KB). It is used for storing executable codes and accumulated values for a small period of time. However, it consumes most of the chip area and much of the power budget. Therefore, a larger amount of extra flash memory, perhaps more than a megabyte, is used on a separate chip to support the enhanced network functionality. The required amount of power can be obtained from many sources. Most sensors deploy a set of AA batteries and/or solar panels [13]. However, in most cases the choice of correct energy source is application specific.

### 2.2. Overview of Flash Memory

Flash memory is a non-volatile solid state memory which has many attractive features such as small size, light weight, fast access speed, shock resistance, high reliability, and low power consumption. Because of these attractive features and decreasing price and increasing capacity, flash memory is becoming ideal storage media for mobile and wireless devices [14].

**Figure 2.**
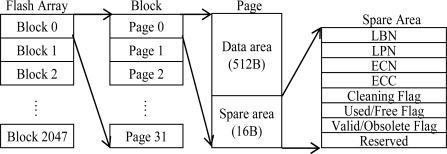
Flash memory (32 MB) architecture.

Flash memory array is partitioned into equal size erase units called blocks and each block is composed of a fixed number of read/write units called pages (Figure 2). Every page has two sections, data area and spare area. Spare area stores metadata like logical block number (LBN), logical page number (LPN), erase count number (ECN), error correction code (ECC), cleaning flag for indicating garbage collection process in block, used/free flag to show page is used or still free, and information of being valid/obsolete about data in data area. The sizes of pages and blocks differ by product.

Flash memory has three kinds of operations: page read, page write, and block erase. The performance of three kinds of operations is summarized based on memory access time and required energy at maximum values as shown in Table 2 [15].

Even though flash memory has many attractive features, its special hardware characteristics impose design challenges on storage systems. It has two main drawbacks:

*First Drawback*: An inefficiency of in-place-update operation. When we modify data, we cannot update data directly at the same address due to the physical erase-before-write characteristics of flash memory. Therefore, updating even one byte data in any page requires an expensive erase operation on the corresponding block before the new data can be rewritten. To address this problem, the system software called flash translation layer (FTL) was introduced, as in [16–18]. FTL uses a non-in-place-update mechanism to avoid having to erase on every data update by using logical-to-physical address mapping table maintained in main memory. Under this mechanism, the FTL remaps each update request to different empty location and then the mapping table updates due to newly changed logical-to-physical addresses. This protects one block from being erased per overwrite. The obsolete data flagged as garbage which a software cleaning process later reclaims. This process is called garbage collection, as in [19–21].
*Second Drawback*: The number of erase operations allowed to each block is limited like10,000 to 1,000,000 times and the single worn-out block affects the usefulness of the entire flash memory device. Therefore, data must be written evenly to all blocks. This operation is named as wear-leveling, as in [22,23]. These drawbacks represent hurdles for developing a reliable flash memory based sensor storage systems.

## 3. PIYAS: Proposed Memory Management Scheme

In this section, we discuss the key approaches of our present research. First, we discuss the sensor data accumulation and buffering in main memory and then data organization in flash memory blocks. Second, we structure the small mapping information by considering the limited SRAM constraints in write intensive conditions. Third, we show how to process the query to access the data from flash in read intensive conditions. Further, we present the garbage collection and wear-leveling policies.

### 3.1. Data Organization Framework

Sensor network storage workload may be highly write-intensive in different scenarios [11]. Data may add more frequently to than it is read from flash memory. We aim to provide an efficient data storage method by sampling data while buffering data in SRAM. Then flash space allocates based on sensor data forms as raw data and aggregate data.

#### 3.1.1. Data Buffers Management

SRAM provides the opportunity to reserve the data buffers to put together the currently accumulated sensor readings from environment and then data store in a sensor’s local memory. Data buffering saves the flash space and reduces the write overhead. We reserve the data buffers by the number of business rules where every buffer size is of one read/write unit of flash memory. When data arrives in the range of any rule, the main memory space assigns dynamically chunks of bytes as buffer. Data of a complete buffer flush in flash memory when the buffer becomes full.

*Business Rules*: Every network has business rules to achieve some business goals. To achieve services in sensor networks, business rules are an effective method for programming a file system for sensor nodes. Rules are logically linked as chain where the structure of rules represents the simple business logic in a compact and efficient way. For example, the business goal says to collect the temperature readings in discrete range from 1F to 80F. In that case, we can split the range in set of rules like (A: [1–20]), (B: [21–40]), (C: [41–60]), (D: [61–80]). The formulation of set of rules highly depends on the probability of type of data accumulation from environment and location for implementation of sensors. Since the sensor nodes assist the real life processes, the variation in set of rules is expected to address the monitoring of service parameters. Therefore, we assume that the set of rules is available to sensor nodes from the network applications.

**Figure 3.**
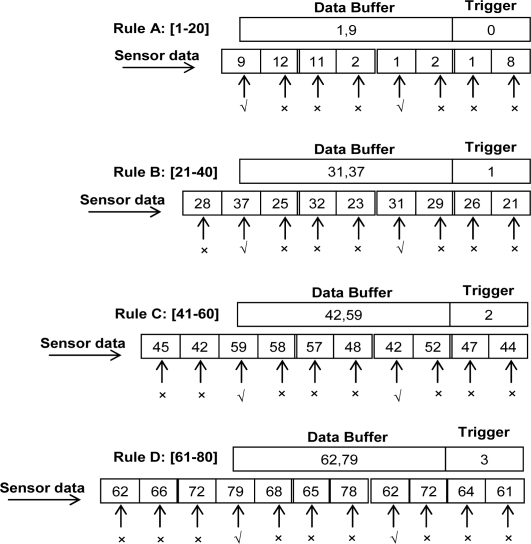
Data buff.ers management in SRAM.

*Example*: We explain our data sampling scheme by an example. In Figure 3, the randomly taken sensor values are continuously coming and buffering with corresponding rules. The trigger option with every data buffer is a fixed threshold to define the gap of the number of times between every value of data. For instance, if the latest value is kept in one buffer then the next value adds in same rule buffer after the number of times of a threshold. Here we set the trigger threshold value as 3. Initially the trigger value is zero. See Rule-A, when the first three values 8, 1, 2 arrive, they only increase the trigger value but are not stored (×) in buffer. However, when a fourth value 1 comes in same Rule-A, the value stores (√) in buffer and the trigger resets to zero again. In the same way, further values 2, 11, 12 are discarded and 9 stores in the buffer. This process repeats until the data buffer becomes full and flushes in flash memory. Data is stored in data buffers for all other rules in the same way.

The main idea behind this sampling approach is to minimize the write overhead in flash when data repeats for same range of values in same period of time, and buffering minimizes the read overhead as well against the single value storage in a single page of memory. The sampling approach also protects against the repeated erases in cases when memory becomes frequently exhausted. The value of sampling threshold may be set higher if user demands more abstract data, otherwise the data can be made concrete by using fine threshold values.

#### 3.1.2. Memory Block Organization

After sampling and buffering the data, we propose to store the sensor data in flash memory in two most user demanding forms as raw data and aggregate data. The raw data are the readings the sensor collects periodically from the environment at regular incremental intervals of time or when some event occurs. Therefore, the raw data blocks (RDBs) individually assign to every rule in the form of a chain. When flash space becomes exhausted, the system selects the victim block for garbage collection from RDBs based on the long chain of blocks and the age of data (see Section 3.4). In conventional schemes, data are simply erased and then the future queries cannot access such data, as in [6–10]. This generates a data failure for the network application. In this paper, instead of permanently discarding data from victim block, the data has second chance to be used for applications. The data from victim RDB aggregate on the most user demanding parameters and are stored in an aggregate data block (ADB).

*Data Aggregation:* We congregate the values of the victim block from the flash erase unit to a read/write unit where every erase unit is composed of multiple read/write units. It means that the number of pages of victim block aggregate based on user defined parameters like MIN, MAX, AVERAGE, COUNT, etc. on single page size. Therefore, every page on the ADB represents the major information of data of one complete previously erased RDB.

**Figure 4.**
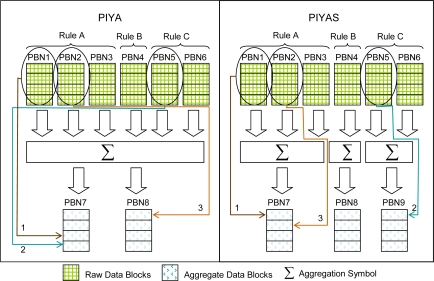
Flash memory block organization.

*Example*: We define our scheme of data organization in memory blocks by an example. Figure 4 shows two sections, our previous scheme *PIYA* and our current enhanced scheme *PIYAS*. We assume that in this example every block consists of four pages.

In the figure, there are three rules: Rule-A, Rule-B and Rule-C, and every rule has separate RDBs allocated. As Rule-A keeps three blocks, physical block number 1 (PBN1, PBN2 and PBN3), Rule-B acquires one block PBN4, and two blocks PBN5 and PBN6 are allotted to Rule-C. Our previous scheme *PIYA* [1] proposed that whenever some RDB selects a victim to erase as there are three blocks they are erased in sequence, first PBN1, second PBN5 and third PBN2, than the combined ADBs PBN7 and PBN8 are used to store the aggregated data from all rules. As in the figure, aggregated data of PBN1 and PBN5 are stored on the last two available pages of PBN7 where we assume that the first two pages are already filled and aggregated data of PBN2 are saved on first available page of PBN8. In that case, search and access of desired data while query responding from ADBs takes long time because data from all rules are saved together. Therefore, *PIYAS* enhances the scheme and proposes to dedicate separate ADBs as PBN7 to Rule-A, PBN8 to Rule-B and PBN9 to Rule-C individually to provide efficient query responses in read intensive scenarios. Therefore, the data aggregation saves space and provides a high availability of in-network data for a long period of time. The data from ADBs is deleted after a user defined time threshold that indicates how long some data should be kept in memory.

### 3.2. Mapping Structures Management

Flash memory mapping information stores in flash media in dedicated map blocks for fast initialization of system. At the time of system startup, the mapping information fetches in SRAM. Limited SRAM and lengthy initialization time are challenging constraints of sensor resources. Therefore, we aim to achieve instant mounting with very small size of SRAM footprints. In our scheme, data is saved sequentially on the first available page of the latest allocated RDB according to some rule. Therefore, every rule keeps only first available physical page number (PPN) in SRAM where single page mapping reserves only 2 bytes in main memory for 32 MB of flash memory which has 2^16^ total number of pages. Therefore, we need only the limited number of pages mapped by the number of rules.

**Figure 5.**
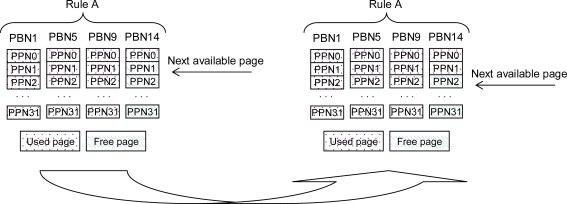
Sequential incremental page allocation.

*Example*: We define our mapping structure by an example. Table 3 shows the mapping structure in SRAM. We assign four rules (A, B, C and D) where every rule takes a temperature value in incremental order. Every rule keeps the first available page number from its latest allocated RDB. Table 4 shows the metadata of the chain of RDBs and ADBs allocated to rules. It describes that the PPN1 of Rule-A in Table 3 belongs to newly allocated RDB PBN14 of Rule-A as PBN14 is shown lastly allotted in chain of blocks of Rule-A in Table 4. The same situation occurs for all other rules. A new write operates sequentially on the first available page from RDB. As Figure 5 shows the RDBs allocated to Rule-A and first available free page as PPN1 of PBN14 that increments by one to PPN2 after write operation. Similarly, memory address increments by the number of pages in one block, automatically.

The meta-data updates in flash map blocks only when the new block allocates to it or an old block deletes from the chain of any rule. It saves the write operations in map blocks by the number of pages in a block minus one.

During initialization, the system reads the last allocated PBNs of RDBs to every rule from the metadata in map blocks and extracts the first available free page addresses for building the mapping table in SRAM. Mounting the information of ADBs in a write intensive scenario is not beneficial because the system needs the information of the ADBs either at the time of garbage collection to save the aggregated data from victim RDB to ADB or in read intensive conditions. In the first condition, the system simply reads the PBN of ADB assigned to the victim block rule and stores the aggregated data on the first available free page. Under the latter condition, the system fetches the complete metadata of RDBs and ADBs by a single commit read operation to entertain the queries effectively.

### 3.3. Query Processing Framework

There are two sensor node states: active and sleep. A sensor node sleeps for a long time to preserve energy and only activates to accumulate data from the environment in predefined time intervals. In this paper, we filter data in data buffers in main memory assigned to every rule and flush in flash memory when data buffers become full. Every data buffer is of same size as the read/write unit of the flash memory so flushing consumes one flash page every time. Every flash memory page has two sections: data area and spare area. The spare section is used to store the metadata regarding the data in the data section. To provide efficient query processing, we store rule based data physically and time based hierarchically. Therefore, while writing data in a data area the related timestamp is also recorded in the spare area of each page. This results in time units for the number of pages in one block.

In the read intensive scenario, our previous scheme *PIYA* extracts the timestamps by reading the spare area of the first page of every block and sets the time between two consecutive RDBs of same rule chain. Then the table arranges in main memory for fast access of data. When some query comes in the range of some rule, the system forwards that in the corresponding block according to the desired time range of the query. The system evaluates the timestamp written in the spare area from the latest written page. If the page supports the queried value, then the system checks the data items inside the data section of page, otherwise it moves one page up.

**Figure 6.**
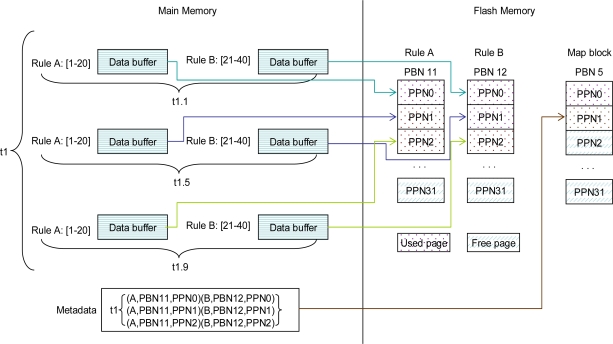
Metadata storage on flash map blocks.

In the case of a large size of space being occupied the scanning by *PIYA* of the spare area of the first page of every block to build the mapping table and then finding the exact pages by reading the spare areas of every page in the corresponding block consumes a long time and high energy. Therefore, in this paper, *PIYAS* implements a more energy efficient data access and provides a high throughput for responses to user queries. We propose to maintain the data storage log in the form of metadata in the dedicated map blocks separate from the file system mapping information. We store the metadata regarding the memory assigned to every rule in a particular time interval as shown in Figure 6, where Rule-A and Rule-B consumes six pages, three for each in their corresponding blocks as PBN11 and PBN12 in time *t1* and at the same time metadata regarding consumed flash pages in time *t1* stores in map block as PBN5 on first available free page as PPN1.

The system fetches the metadata from map blocks and builds the query processing framework in main memory for entertaining the read intensive scenarios efficiently. Table 5 presents the logical framework of memory where all data are sequentially stored in time hierarchy but actually divided in rules. The table shows the physical addresses accumulated by sensor raw data in every timestamp and empty spaces show that no data was collected by the sensor for rules in such time frames. We gather the metadata like timestamp as from *t1* to *t6* where *t1* is oldest and *t6* is latest timestamp and the assigned PBN and its corresponding PPNs regarding every individual rule.

For addressing the queries on already aggregated data, we keep only PBNs of ADBs in main memory like *PIYA*. Therefore, we have advantage that our scheme preserves the energy and takes a reduced search time for answering any query because we allocate the separate ADBs to individual rules, see Table 5. Therefore, unlike the *PIYA* scheme, *PIYAS* does not have to read the spare areas of pages of unconcerned rules.

#### 3.3.1. Query on Raw Data Blocks

We explain the time-based, value-based and hybrid queries in detail in the following paragraphs. However, the comparison and aggregation based queries follow the same way to extract data from flash media. For better understanding of query processing framework in following examples, we refer to Table 5.

*Time-Based Queries:* Time-based or temporal query answers based on the evaluation of timestamps recorded by every physical address to satisfy a given situation. Queries like: *find the five latest records of maximum temperature?* are answered by scanning the latest records of timestamp *t4* of *Rule-D*: [61–80] because Rule-D keeps the values of the range of maximum temperature. The system reads the lastly allocated PBN and its corresponding latest written PPNs as *14*(*3,4,5*) and extracts the data directly from specified physical addresses. It takes only three read operations and protects from unnecessary reads.

*Value-Based Queries:* Value-based queries are answered by evaluating the range of data required. A query like: *find the latest records of temperature between 50F–70F?* clearly shows the range belongs to *Rule-C*: [41–60*]* and *Rule-D*: [61–80]. The value answers by scanning the latest readings of both rules as *13*(*6,7,8*) and *14*(*3,4,5*), respectively. It takes only six read operations to effectively respond to the query.

*Hybrid Queries:* Hybrid queries are combinations of time-based and value-based queries. Queries like: *find five records of temperature between 25F–35F in time t2?* are answered by reading the values from *12*(*3,4,5*) of timestamp *t2* from *Rule-B*: [21–40]. It takes only three read operations.

#### 3.3.2. Queries on Aggregate Data Blocks

If the queried time unit is less than the oldest timestamp available in RDBs, the system forwards the search query on to ADBs. For example, a query like: *find five records of max temperature at time t1-1?* denotes that queried time period *t1-1* is less than the least timestamp *t1* available in raw data, although *t1-1* ∈ *ADB* as it is available in the range of aggregated data. So the query transfers to the dedicated ADB of the corresponding rule.

In our previous scheme *PIYA* that system evaluated both the rule symbol and timestamps written in the spare area. If the rule supports the queried value then the system checks the data items inside the data section of page, otherwise it moves one page up. In that case, for answering any query, the system may have to attempt many read operations unnecessarily as system previously used the combined ADBs for all rules.

In this paper, we assign separate ADBs to every rule, which minimizes the read overhead for accessing the required data. We adopt the same procedure to respond to any query but it becomes more simple and efficient to forward a query direct to the exact ADB of the queried range rule. As above the query is answered by scanning the records of ADB as PBN23 in Table 5 of *Rule-D*: [61–80] because Rule-D stores the values of the range of maximum temperature. The system evaluates the timestamp written in the spare area from the latest written page for answering temporal queries. If a page supports the queried value, it extracts the data items inside the data section of page otherwise it moves one page up.

### 3.4. Garbage Collection

Under real workload conditions, thousands of readings are stored in flash memory and accessed in different situations. All data is stored sequentially and may be accessed randomly depending on user demand. Data has its user defined in-network life and after a predefined time threshold data becomes dead and the block keeping dead data is marked obsolete. To make space for new data, the reclamation takes place either in the background when the system is idle or on-demand when the amount of free space drops below some predetermined threshold.

In this paper, the first priority for erasure is for already obsolete blocks. We achieve reduced erase operations as well as efficient wear-leveling by not erasing blocks immediately after they become obsolete. Blocks are collected in dirty blocks pool and when system triggers the cleaner for free space, the block with the least erase count number (ECN) is selected for erasure and then provided for new data. The ECN is the meta-information used to keep the erase record of a block. In another case, if there is no obsolete block available, the victim block selection is applied by following steps:

1. *Step 1*: Select rule(s) with long chain of raw and aggregate data blocks. Every time, for cleaning the system evaluates two blocks from the long chain of blocks, one from RDBs and other from ADBs. The system selects the blocks by a first-in-first-out policy. It means the block with the oldest timestamp is always selected for erasure. RDB and ADB can be selected by the same or different rules, depending on the long chain of blocks in any rule.
2. *Step 2*: Evaluate selected ADB. The system evaluates the timestamp of the last written page of the oldest ADB. If the data is dead, meaning the timestamp crossed the threshold of allowed in-network data sustainability, then the system marks the whole block as obsolete. As the last written page of every block represents the latest data within the block, if the last written page data becomes dead, then by default the data in all the previous pages become dead too. Then the block is erased and made available for new data. In the other case, if a block is still alive then the system goes to step three.
3. *Step 3*: Evaluate selected RDB. The system evaluates the timestamp of the last written page of the oldest RDB. If the data exceeds the life limit then the system erases the block and provides it for new data. In the other case, the system evaluates the timestamps recorded in the spare areas of every page according to the time threshold of in-network data sustainability. The pages from the block under observation that are still alive aggregate on the user provided aggregation parameters. The system aggregates the data from the block size to page size and rewrites the aggregated data in the first available free page of ADB dedicated to the corresponding rule. Finally, the victim block is erased and becomes available for new data.
4. *Step 4*: Perform cross checking for data sustainability periodically. The system evaluates the last written pages of the oldest blocks from the rules those have not participated in cleaning operations for a long time because they may not have long chains of blocks. The system considers both raw and aggregate data blocks. If the data is still alive, the system retains them or else blocks are marked obsolete and considered part of the dirty blocks pool. The system erases such blocks when idle.

### 3.5. Wear Leveling

A good wear-leveling policy evenly distributes the erase cycles on all blocks to prolong the life time of flash media. Thus the effectiveness of a wear-leveling policy could be evaluated in terms of the standard variation of erase counts of all blocks and the earliest appearance time of the first worn-out block. To evenly wear down the flash media, we allocate free blocks by their ECN. We assign low ECN free blocks for RDBs and high ECN free blocks for ADBs. It is because the ADBs experience comparatively more stay time in flash media than RDBs. As the data in RDBs aggregate and are saved on ADBs therefore they have more probability to be selected as victims for cleaning than blocks holding aggregate data. On the other hand, the blocks with aggregate data hold the data for long time intervals as each and every page of ADB represents the timestamp of a previous complete RDB. Therefore, they live long in memory and can only be marked obsolete when the data on all pages of the ADB becomes dead.

## 4. Performance Evaluation

### 4.1. Simulation Methodology

To demonstrate the performance effectiveness of our proposed *PIYAS* scheme, we performed a trace based simulation. We compare *PIYAS* with previous schemes such as *PIYA* [1] and MicroHash [10]. Evaluation focuses on four parameters:

- *Space Management*: This shows the flash memory allocation against the thousands of continuous sensor readings and main memory consumption for maintaining the data buffers and metadata.
- *Search Performance*: This shows the number of pages required to be read for responding to a query.
- *Throughput Performance*: This shows the response of number of queries in a unit of time.
- *Energy Consumption*: This shows the energy consumption while data writes to and data is read from sensor local flash memory.

We have built a simulator with 32 Megabytes of flash space that is divided into erase blocks of equal size. Each block size is 16 kilobytes and every block is composed of 32 pages as read/write units. Every page size is 512 bytes with 16 bytes spare area. We extracted the trace file from *COAGMET* [24]. The two years raw data were extracted on an hourly basis from January 01-2007 to December 31-2008 from the Willington climate station. The trace file contains a total of 279,968 sensor readings and it is a combination of all known data formats like negative, positive and decimal values. To prove the enhancement of our idea for large size of sensor data centric applications, we experimented with a broad range of rule values. Rules are adopted as directory buckets in the case of MicroHash. The rules are given in Table 6.

The total elapsed time is calculated by Equation (1) for effective comparison between schemes. Time required for read in unit of page from flash memory to data register is calculated by Equation (2). Time required for read a byte unit from the data register to main memory is calculated by Equation (3). Time required for computation in main memory for building the mapping structure and the query processing framework is calculated by Equation (4). Time required to write data from the main memory to flash media is calculated by Equation (5). For better understanding of experimental results in terms of time and energy, we refer to Table 2.

(1) $$\mathrm{Total time}\;={TR}_{\mathrm{FR}}+{TR}_{\mathrm{RR}}+T\alpha +{TW}_{\mathrm{RF}}$$

(2) $${TR}_{\mathrm{FR}}=\left(\mathrm{read}\;\mathrm{count}\left(\mathrm{page}\right)\times \mathrm{read}\;\mathrm{time}\right)$$

(3) $${TR}_{\mathrm{RR}}=\left(\mathrm{read}\;\mathrm{count}\left(\mathrm{byte}\right)\times \mathrm{read}\;\mathrm{time}\right)$$

(4) Tα=Time for Computation in RAM

(5) $${TW}_{\mathrm{RF}}=\left(\mathrm{writecount}\left(\mathrm{page}\right)\times \mathrm{writetime}\right)$$

### 4.2. Experimental Results

**Figure 7.**
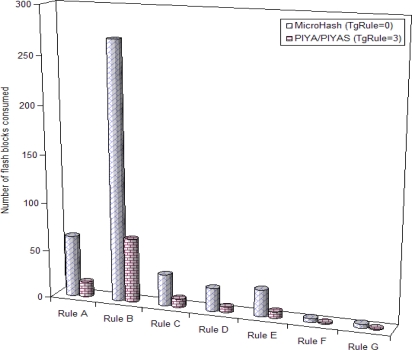
Flash memory consumption in number of erase units.

Figure 7 shows the consumption of flash memory in number of erase blocks for number of sensor readings attempted by every rule. The trigger with every individual rule buffer (TgRule) is used in SRAM for sampling the sensor readings. We show the fine granularity of data arrival in buffer of every rule by taking a small value of threshold as TgRule = 3 for *PIYA* and *PIYAS* schemes and as MicroHash does not sample the data so we show the consumption of media for MicroHash by keeping the trigger unset as TgRule = 0. In the figure, the flash blocks are individually allocated as chains to every rule for saving the sensor data corresponding to a trigger threshold where thousands of readings are stored in a very small flash memory space by both the *PIYA* and *PIYAS* schemes. MicroHash stores data in linear sequential order. Therefore, we calculated the blocks consumed by MicroHash by counting the number of pages allotted to every bucket. In this result, we only show the space consumed by data pages and space assigned to metadata is not added. However, results clearly show the effectiveness of our memory management scheme. Our proposed schemes outperform the MicroHash for efficient media utilization.

**Figure 8.**
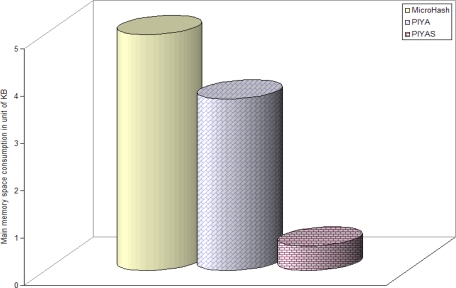
SRAM consumption in KBs for buffering sensor readings.

Figure 8 shows the consumption of SRAM space in kilobyte units while the sensor filters and buffers the accumulated readings. Results show that the proposed *PIYAS* scheme clearly outperforms both the *PIYA* and MicroHash schemes. This is because unlike *PIYA* and MicroHash, *PIYAS* does not allocate static buffers but buffers are alloted dynamically in chunks of bytes whenever some sensor reading arrives in the data buffer of some rule. Therefore, even though in a very write intensive scenario, *PIYAS* optimizes main memory space accumulation by 71.4% and 79.2% more than *PIYA* and MicroHash, respectively.

**Figure 9.**
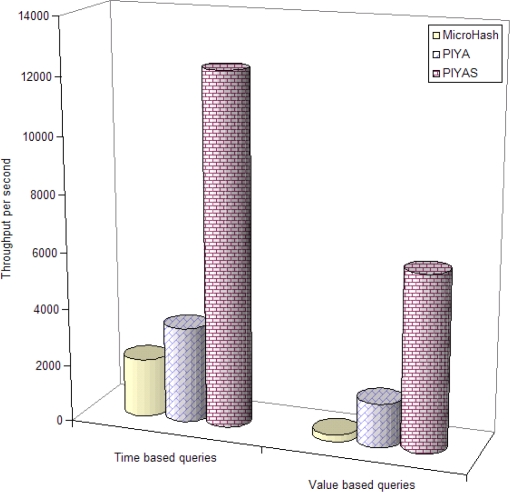
Data throughput per second from RDBs.

**Figure 10.**
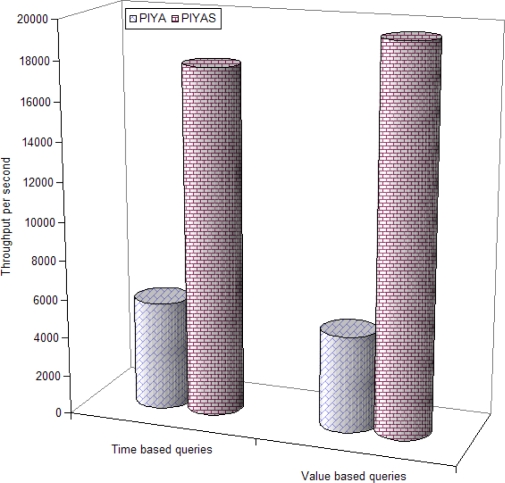
Data throughput per second from ADBs.

In a read intensive scenario, the *PIYAS* scheme requires 3.57 and 7.56 times more space in SRAM and 56.7% and 6.9% more time while building the query processing framework, compared to *PIYA* and MicroHash, respectively, but *PIYAS* greatly outperforms both the previous schemes in time required for query responses. Figures 9 and 10 show the time based and value based query throughput by the average number of queries responded per second time unit from raw and aggregate data blocks, respectively. We did not experience any erase operations on RDBs. It is because we set the threshold of free space at two blocks. This means that the first erase operation is performed when the memory is completely filled and only two blocks remain free. Therefore, for evaluating the ADBs we aggregated the raw data and composed three pages for each rule.

Results in Figures 9 and 10 show the effectiveness of our enhanced memory management scheme. *PIYAS* gives a highly improved throughput compares to both previous schemes. Results observed from ADBs are particularly encouraging. This is because *PIYAS* does not need to read the unnecessary pages of unconcerned rules from ADBs to answer a query as *PIYA* does. Figure 10 only presents the results from the *PIYA* and *PIYAS* schemes, and MicroHash is not included because when memory exhausts MicroHash simply erases the data and then network applications cannot access such data for future queries, which results the data failure. However, the *PIYA* and *PIYAS* schemes effectively aggregate such data and store it in ADBs for long-term in-network data availability.

For our simulation, we composed sensor values in seven rules, see Table 6. At system initialization time, for building the mapping table, we extract the mapping information from map blocks to the main memory. The metadata in map blocks holds the definition of rules and the PBNs assigned to every rule. The definition of every rule takes 20-Bytes of space and every rule requires an address of one page for mapping information in main memory, where every page is mapped by 2-Bytes for 32 MB flash memory which thus has 2^16^ total pages. We obtain a fast mounting in 136.75 μs; it consumes 0.396 J and 154-Bytes in SRAM. Therefore, both the *PIYA* and *PIYAS* schemes use the same time and number of bytes while mounting the mapping structure in main memory and for saving the mapping information back to the map blocks.

Table 7 shows the resources optimization by the *PIYAS* scheme compared to both previous schemes. This information is calculated by obtaining the results of average number of pages system reads on every request from network applications while searching the queried data in a very read intensive environment by all three schemes. For better understanding the results in terms of time and energy, we refer to Table 2. The experimental results show that compared to *PIYA*, *PIYAS* saves 216.4 μs time plus 0.95 J energy and 120.9 μs time plus 0.53 J energy for time based queries, 487.2 μs time plus 2.14 J energy and 150.5 μs time plus 0.66 J energy for value based queries from RDBs and ADBs, respectively. Compared to MicroHash, *PIYAS* saves 406 μs time plus 1.79 J energy for time based queries and 3897.6 μs time plus 17.15 J energy for value based queries from RDBs. MicroHash does not support aggregate data so the results are not applicable (N/A) from ADBs for MicroHash.

Table 8 shows the throughput optimization by *PIYAS* scheme. Compared to *PIYA*, *PIYAS* achieves 72.7% and 68.7% time based, 75% and 74.6% value based more queries per second throughput from raw and aggregate data blocks, respectively. Compared to MicroHash, *PIYAS* obtains 83.3% time based and 96% value based more queries per second throughput from RDBs.

In our experiment, although we did not experience any erase operation, since our proposed garbage collection and wear-leveling schemes are particularly well designed for sensor relevant memory management environment they should definitely perform well.

## 5. Conclusions

This research proposed a novel log-structured external NAND flash memory based data management scheme called *Proceeding to Intelligent service oriented memorY Allocation for flash based data centric Sensor devices in wireless sensor networks (PIYAS)*. We achieved instant mounting and reduced SRAM footprints by keeping a very low mapping information size. The main memory required for accumulation of sensor readings is minimized. We optimized storage utilization by effective data buffering in main memory before writing data to flash media. Data failure is mitigated by long term in-network data availability. We optimized the throughput of query responses by allocation of memory blocks individually on the basis of predefined business oriented rules. Fast access of memory to write data, computation *in situ*, high query throughput, more energy efficiency and minimized reads, writes and erases are effectively achieved. Sensor environment oriented garbage collection and wear-leveling schemes are also employed. We performed trace driven simulations to explore in detail the effectiveness of our idea. Our comprehensive experimental results with real traces from environmental and habitat monitoring show that *PIYAS* is an optimized memory management scheme for modern wireless sensor devices.

## Figures and Tables

**Table 1.** Comparison of PIYAS with previous schemes.

**Technique**	**Storage Device**	**Storage Location**	**Data Life Efficient**	**Device Life Efficient**	**Energy Efficient**	**Storage Efficient**
Matchbox	NOR	Internal	No	No	No	No
ELF	NOR	Internal	No	Yes	No	No
Capsule	NOR/NAND	Internal	No	Yes	Yes	No
MicroHash	NAND	External	No	Yes	Yes	No
PIYA	NAND	External	Yes	Yes	Yes	Yes
PIYAS	NAND	External	Yes	Yes	Yes	Yes

**Table 2.** Performance of NAND flash memory.

**Operation**	**Time (μsec)**	**Energy (mA) (Current 3.3 V)**
Page Read (512 + 16) B	15	20
Page Write (512 + 16) B	500	25
Block Erase (16K + 512) B	3,000	25

**Table 3.** Mapping table in SRAM.

**Rule Description**	**PBN (PPN)**
A: [1–20]	14(1)
B: [21–40]	15(2)
C: [41–60]	16(3)
D: [61–80]	17(4)

**Table 4.** Metadata in flash map blocks.

**Rule Description**	**PBN (Raw Data)**	**PBN (Aggregate Data)**
A: [1–20]	1,5,9,14	13
B: [21–40]	2,6,10,15	-
C: [41–60]	3,7,11,16	18
D: [61–80]	4,8,12,17	-

**Table 5.** Query processing framework.

**Rule Description**	**A: [1–20]**	**A: [21–40]**	**A: [41–60]**	**A: [61–80]**

**Timestamp**	**PBN (PPN)**	**PBN (PPN)**	**PBN (PPN)**	**PBN (PPN)**

**t1**	11 (0,1,2)	12 (0,1,2)	-	-
**t2**	-	12 (3,4,5)	13 (0,1,2)	-
**t3**	-	-	13 (3,4,5)	14 (0,1,2)
**t4**	11 (3,4,5)	-	-	14 (3,4,5)
**t5**	11 (6,7,8)	12 (6,7,8)	-	-
**t6**	-	12 (9,10,11)	13 (6,7,8)	-
**Aggregate Data Blocks**	21	-	22	23

**Table 6.** Rules description for simulation.

**Rule Symbol**	**Rule Range (Temperature)**
A	−99–0
B	1–100
C	101–200
D	201–300
E	301–400
F	401–500
G	501–600

**Table 7.** Resources (Time and Energy) preservation by PIYAS. Comparison to PIYA and MicroHash.

			*PIYA*	*MicroHash*
RDBs	Time based queries	Time Energy	216.4 μs 0.95 J	406 μs 1.79 J
	Value based queries	Time Energy	487.2 μs 2.14 J	3897.6 μs 17.15 J
ADBs	Time based queries	Time Energy	120.9 μs 0.53 J	N/A N/A
	Value based queries	Time Energy	150.5 μs 0.66 J	N/A N/A

**Table 8.** Throughput per Second Optimization by PIYAS. Comparison to PIYA and MicroHash:

		*PIYA*	*MicroHash*
RDBs	Time based queries Value based queries	72.7% 75%	83.3% 96%
ADBs	Time based queries Value based queries	68.7% 74.6%	N/A N/A
